# Mechanisms of the Blunting of the Sympatho-Adrenal Response: A Theory

**DOI:** 10.2174/157339909788166846

**Published:** 2009-05

**Authors:** B Parekh

**Affiliations:** Institute of Metabolic Science, University of Cambridge, Cambridge CB2 0QQ, UK

**Keywords:** Hypoglycaemia unawareness, Insulin therapy, Defective arousal, Glucose-sensing, Brain glycogen, Noradrenaline.

## Abstract

Development of therapeutic measures to reduce the risk of potentially fatal episodes of hypoglycaemia and thus to achieve the full benefits of intensive insulin therapy in diabetic patients requires a complete understanding of the multifactorial mechanisms for repeated hypoglycaemia-induced blunting of the sympatho-adrenal response (BSAR). After critical analysis of the hypotheses, this review paper suggests a heuristic theory. This theory suggests two mechanisms for the BSAR, each involving a critical role for the central brain noradrenergic system. Furthermore, this theory also suggests that the lateral hypothalamus (LH) plays an important role in this phenomenon. Within the framework of this theory, explanations for 1) sexual dimorphism in the adrenomedullary response (AR), 2) dissociation in the blunting of the AR and the sympathetic response (SR) and 3) antecedent exercise-induced blunting of the AR are provided. In addition, habituation of orexin-A neurons is suggested to cause defective awakening. Moreover, potential therapeutics measures have been also suggested that will reduce or prevent severe episodes of hypoglycaemia.

## 1 INTRODUCTION

In insulin-treated (T1DM and advanced T2DM) diabetic patients the sympatho-adrenal response (SAR) is the main defence against hypoglycaemia. This is because of the lack of a first-line counter-regulatory response [1, 2]. The SAR includes the adrenomedullary response (AR) and the sympathoneural response (SR) [3]. The AR involves an adrenomedullae-mediated epinephrine release that causes hyperglycaemia [1, 2]. The SR involves release of noradrenaline and acetylcholine, which causes adrenergic symptoms (shaking and tremors) and cholinergic symptom (sweating) respectively [1, 2, 4]. These symptoms prompt patients to take actions against hypoglycaemia. 

However, due to technological limitations, injections of inappropriate insulin doses cause frequent episodes of mild hypoglycaemia which lead to the blunting of the SAR (BSAR) [1, 5]. The blunting of the AR, which causes defective glucose counter-regulation, alone increases the risk of severe hypoglycaemia by a factor of 25 [1]. The BSAR, which involves the blunting of the AR and SR, causes both defective glucose counter-regulation and the loss of the external symptoms, leading to hypoglycaemia unawareness (HU) [1]. HU increases the risk of suffering from severe and potentially fatal episodes of hypoglycaemia [1]. Hence, insight into mechanisms which causes the BSAR and leads to HU could provide measures that will reduce the risk of potentially fatal hypoglycaemia, thereby allowing patients to receive the full benefits of insulin therapy such as reduction in micro- and macrovascular complications. Indeed, the search for the mechanisms has been the focus of intensive research for well over four decades [5]. However, a complete understanding of the mechanisms involved still remains elusive. This paper suggests a new theory which proposes two mechanisms involved in the BSAR, each involving the role of central noradrenergic neurons. 

This theory has been developed in three steps. First, through critical examination, promising hypotheses have been extracted from previously proposed hypotheses. Second, through literature review, neurotransmitters, neural pathways and brain structures involved in mediating the SAR have been synthesised. Third, these pathways and promising hypotheses have been integrated to suggest a heuristic theory that could satisfactorily explain the BSAR. In this paper, this theory is also described in these logical steps. Additionally, explanations for various observations (i.e. sexual dimorphism) related to the BSAR are also provided. Finally, therapeutic implications, arising from this theory, are discussed. The paper ends with conclusion and summary. 

## 2 REVIEW

Researchers, so far, have proposed five major hypotheses to explain the BSAR. These are (1) the systemic mediator hypothesis [1], (2) the increase in brain fuel transport hypothesis [1], (3) the brain glycogen super-compensation hypothesis [1], (4) reduction in the sensitivity of the glucose sensing neurons (GSNs) in the VMH, and (5) the role of cortical and thalamic structures. 

Davis and colleagues proposed the systemic mediator hypothesis which suggests a role of cortisol in the BSAR [1]. The increased brain fuel (glucose) transport hypothesis suggests that an increased brain glucose supply caused by antecedent hypoglycaemia blunts the SAR and causes unawareness of hypoglycaemia [1]. These two hypotheses have been discussed critically before in an excellent review paper [1]. Therefore, no further discussion is warranted in this review. In short, experimental evidence suggests that the proposed factors suggested by these hypotheses seem unlikely to blunt the SAR. 

### 2.1 Increased Brain Fuel (Monocarboxylate) Transport Hypothesis

This hypothesis suggests that increase in the supply of brain monocarboxylase fuels (lactate, fatty acids) plays a role in the BSAR [1]. Earlier research showed that infusion of ketones and non-glucose lipids shifted the threshold for epinephrine secretion (but not glucagon), delayed its secretion, and reduced its quantity [6, 7]. Researchers further demonstrated that lactate infusion before or after hypoglycaemia blunts the AR [8, 9]. Thus, these observations strongly suggest the role of these fuels in the blunting of the AR. Of note, along with the AR, these studies showed that the infusion of these fuels blunt cortisol secretion as well [6-9]. On the other hand, findings from these studies are inconsistent for growth hormone and noradrenaline responses. Two of these studies reported that monocarboxylate fuels blunt both growth hormone and noradrenaline [6, 9]; one of them reported that only growth hormone is blunted [7]; and one of them reported no effect on either of them [8]. Nevertheless, with the context of the SAR, this hypothesis can provide only a partial explanation for the BSAR. This is in agreement with observations that suggest at least two separate mechanisms of the BSAR [10, 11]. For example, Davis *et al*. demonstrated that even two short episodes (~5 min) of antecedent hypoglycaemia can blunt the AR while intermediate and prolonged episodes of hypoglycaemia blunts the symptoms of hypoglycaemia, essential for hypoglycaemia awareness [11]. 

### 2.2 Brain Glycogen Super-Compensation Hypothesis

Glycogen is also found in the brain, which is mainly located in the astrocytes [12]. Astrocyte glycogen content is modulated by many factors including neurotransmitters (i.e. NA, serotonin) and arterial glucose concentration [12, 13]. Gruetter *et al*. proposed that brain glycogen plays a role in the BSAR [13, 14]. It is based on evidence from a NMR study which showed that in rats, the brain glycogen levels increased several fold after an antecedent hypoglycaemia [14]. This hypothesis posits that previous exposure to hypoglycaemia raises brain glycogen levels which supplies fuel (lactate) to neurons and leads to the BSAR [13, 14]. Consistent with this hypothesis, Alquier *et al.* have recently also reported accumulation of glycogen in the hypothalamus after an episode of glucopenia which remained elevated for 20 hr in rats [15]. Oz *et al. *further demonstrated that, in healthy volunteers, during moderate hypoglycaemia glycogen is utilised in the human brain [16]. This is in keeping with an observation that mild stress (i.e. immobilisation) in rats causes glycogenolysis and lactate formation from the astrocytes [17]. Conversely, high intensity exercise that causes brain glycogen depletion is associated with exaggeration in the AR in response to hypoglycaemia [18-20], further supporting this hypothesis. In agreement with this, Barnes *et al*. showed that infusion of isofagomine, a glycogen phosphorylase inhibitor, in rats prior to hypoglycaemia in the VMH reduced the availability of both glucose and lactate [21]. Furthermore, they demonstrated that attenuation of glycogen breakdown during the fourth episodes of hypoglycaemia increase the AR and norepinephrine [21]. So, this hypothesis has potential to explain the blunting of the AR. 

However, a recent study calls for refinement of this hypothesis. Herzog *et al. *found that, in rats, after exposure to repeated hypoglycaemia (which was terminated by providing food), only glycogen compensation occurs in the brain and yet the blunting of the AR has already developed [22]. This is in contrast to an earlier study by Choi *et al.* which showed that glycogen super-compensation occurs after an episode of hypoglycaemia [14]. However, experimental design might explain this discrepancy in glycogen levels. Choi *et al*. terminated hypoglycaemia by infusing glucose levels to hyperglycaemic levels whereas Herzog *et al*. terminated hypoglycaemia by providing food. Since arterial glucose concentration modulates glycogen synthesis, this could explain why Choi *et al*. reported glycogen super-compensation. Nevertheless, this suggests that super-compensation of glycogen is not necessary but even normal glycogen compensation might be sufficient to blunt the AR. However, the mechanism of post-hypoglycaemic accumulation of glycogen is not yet established. Moreover, the mechanism of glycogen depletion during subsequent hypoglycaemia and how this depletion might contribute to the blunting of the AR during later episodes remains to be discovered. 

### 2.3 Reduction in the Sensitivity of the GSN in the VMH

The VMH, which includes the arcuate (ARC) and ventromedial nuclei (VMN), contains glucosensing neurons. It has received significant attention with regard to the BSAR [23-26]. It possesses two main types of specialised GSNs: glucose-excited (GE) and glucose-inhibited (GI) neurons [23-26]. GE neurons increase, whereas GI neurons decrease their firing rate as ambient glucose levels rise [23-26]. The VMH has connections to the autonomic outflow areas of the spinal cord [25]. Therefore, it is well placed to detect glucose levels and elicit the SAR. Indeed, a set of earlier studies showed that a lesion of the VMH and local glucose infusion reduces the hormonal response to hypoglycaemia, whereas local 2-DG induced glucopenia stimulates the hormonal responses [27-29]. These studies have led to the notion that recurrent episodes of hypoglycaemia dull the sensitivity of GSNs through as yet to be identified mechanism(s), and consequently cause the BSAR during later episodes [1, 23, 24]. Since then, researchers have sought to discover these unknown mechanism(s). In this regard, several mechanisms have been proposed and significant amounts of data have been collected in support of each. These proposals are reviewed below. 

#### The Role of K_ATP_ Channels

Researchers reasoned that since GE neurons use K_ATP_ channels to sense glucose, alteration in these channels due to antecedent hypoglycaemia plays a role in the BSAR [23, 24]. In support of this suggestion, Evans *et al*. showed that pharmacological K_ATP_ channel blockers (glibenclamide and tolbutamide) reduced both glucagon and epinephrine secretion during both brain glucopenia and systemic hypoglycaemia in rats [30]. In agreement with this study, using a recurrent hypoglycaemia (3 days) rat model, McCrimmon *et al*. showed that K_ATP_ channel openers (diazoxide and NN414) increased the levels of these hormones in both antecedent hypoglycaemia-naive rats and rats with defective hormonal responses induced by recurrent hypoglycaemia [31]. Additionally, by using these pharmacological agents (i.e. diazoxide and glybenclamide), Chan *et al*. showed that K_ATP_ channels-mediated GABAergic modulation could be the mechanism through which K_ATP_ channels modulate these hormonal responses [32]. However, a pharmacological study on humans [33] produced evidence which contradicted these earlier findings. Additionally, Miki *et al*. showed that K_ATP_ knockout mice have defective glucagon response but not the AR [34]. Furthermore, the drugs used in the supporting studies act independently and thus may lack specificity [35, 36]. For example, diazoxide can independently increase glutamatergic currents and reduce GABAergic currents [35]. Therefore, the evidence from these supporting studies is questionable. Collectively, unequivocal evidence is lacking in support of this suggestion. 

#### Lactate Induced Reduction in GI Neuron’s Sensitivity 

In the light of observations that lactate infusion blunts the AR, investigators suggested that the lactate could mediate this effect through altering sensitivity of GI neurons [24, 37]. Song and Routh found that after 3 days of repeated episodes of hypoglycaemia, young suckling rats developed impaired hormonal response against hypoglycaemia [37]. *In vitro* testing of GI neurons from these rats showed that these neurons do not respond to a decrease in extracellular glucose. This was interpreted as an indicator of reduction in their sensitivity. Interestingly, in a simultaneously performed *in vitro *study, they also found that lactate addition also produces the same effect. Based on these observations, the authors inferred that repeated hypoglycaemic-induced increase in lactate release reduces the GI neurons’ sensitivity, thus leading to the blunting of the AR. However, other studies reported conflicting evidence. In contrast, earlier *in vitro* work of Song and Routh showed that GI neurons are excited by lactate in both high and low glucose concentrations [38]. Whereas Yang *et al*. found that lactate addition inhibits the firing rate of GI neurons [39]. Moreover, the inferential nature of these findings from the suckling rat animal model and the technical difficulties in recording the GI neuron’s activity serve as a potential source of inaccuracy [37]. Taken as a whole, unequivocal evidence for this proposition is lacking, raising the possibility that lactate blunts the AR through another independent mechanism. 

#### Alteration in AMPK Activity

Investigators proposed that antecedent hypoglycaemia-induced alteration in AMPK, in GI neurons [23, 24], plays a role in the BSAR. In support of this, McCrimmon *et al*. reported that the administration of drug AICAR within the VMH amplifies the hormonal responses (i.e. glucagon and epinephrine) to acute hypoglycaemia in rats and also in rats with hypoglycaemia-induced defective hormonal responses to hypoglycaemia [40]. Alquier *et al*. also reported that repetitive intracerebroventricular injection of 2-DG in the VMH impair or delay AMPK activation after 3 days [15]. However, the data from the study using AICAR is questionable, as AICAR can independently change glutamate levels and produce the observed effect [15, 41]. Furthermore, the observed delayed activation of AMPK occurred after 3 days, whereas a study reported that even two short episodes (~5 min) of antecedent hypoglycaemia can blunt the AR [11]. Thus, it seems unlikely that alteration in AMPK blunts the AR. 

#### The Role of CRF

McCrimmon *et al. *reported that previous exposure to urocortin I, a CRF-2 receptor agonist reduces the sensitivity of GSNs, which was correlated with the blunting of the AR and glucagon in the later episode [42]. Interestingly, they also showed that this effect lasted for 24 hours. Since the CRF-1 and CRF-2 receptors act in functionally opposite ways, they further showed that the CRF-1 receptor agonist in the VMH, but not in the PVH, stimulates the hormonal responses, whereas the CRF-1 receptor antagonist suppresses them [43]. Nevertheless, previous studies implicated the role of the CRF-1 receptor within the PVH in mediating the SAR [44, 45]. So, it is difficult to reconcile these findings with the earlier studies. Therefore, until the discovery of any concrete causal link, the role of CRF system remains suggestive but not definitive. 

#### Alteration in GK Activity

Researchers suggested that since glucokinase (GK) is the rate-limiting step in glucosensing [23, 46, 47], antecedent hypoglycaemia-induced alteration in GK expression participates in the BSAR. In support of this, *in vitro* investigations showed that by inhibiting GK activity with either RNA interference (RNAi) or with a variety of drugs reduces the sensitivity of VMN glucosensing neurons [46]. Furthermore, an *in vivo* investigation showed that except for fasting, GK expression is up-regulated in rat models (antecedent insulin-induced hypoglycaemia and diet-induced obesity) with defective glucosensing [48]. Kang *et al.* further showed that an episode of insulin-induced hypoglycaemia in rats causes AR blunting in association with augmented GK mRNA expression [49]. Despite this wealth of evidence, which is mainly derived from *in vitro *pharmacological and associative studies, the role of GK in BSAR remains controversial. Fasting– in which GK expression remains unchanged [48]–blunts the SAR to insulin-induced hypoglycaemia [50]. This raises a possibility that GK expression might be modulated by insulin or another unidentified factor rather than hypoglycaemia *per se*. 

Altogether, the notion that changes in the sensitivity of GSNs in the VMH cause the BSAR is not definitively proven. The evidence supporting the proposed hypotheses is either associative or ambiguous but not definitive. Finally, it is still not very clear which specific (i.e. VMN or ARC) nuclei of the VMH participates in the BSAR. Thus, convincing evidence is lacking for the view that GSNs mediate the SAR and that the BSAR occurs due to changes in the sensitivity GSNs through any of these molecular mechanisms. 

### 2.4 Role of Cortical and Sub-Cortical Structures

The complete BSAR may also involve cortical and sub-cortical structures. Using [^15^O] water and positron emission tomography (PET), Teves *et al.* first showed that in healthy volunteers, acute hypoglycaemia activates the OFC, mPFC and rACC as well as the thalamus [51]. Using functional magnetic resonance imaging (fMRI), Musen *et al*. showed that along with the hypothalamus and brainstem, hypoglycaemia activates higher brain regions in both T1DM patients and healthy subjects [52]. 

Since cortical structures regulate the hypothalamus and brainstem [51, 53], Cryer suggested that cortical structures modulate the SAR to lower levels [1]. Relevant to this suggestion, a number of studies reported the differences in the activity of cortical areas between hypoglycaemic aware and unaware T1DM patients. In a [^11^C]-3-*O*-methyl-D-glucose (CMG) PET imaging study, Bingham *et al.* found higher cortical neuronal activation in aware patients than unaware T1DM patients [54]. Using [^18^F]-the fluorodeoxyglucose (FDG) PET imaging technique, Dunn *et al*. found differences in the activation of the OFC, amygdala and hypothalamus between T1DM aware and unaware male patients [55]. 

Recently, using [^15^O] water PET measurements of regional cerebral blood flow Arbelaez *et al.* showed that the BSAR was associated with higher synaptic activity in the dorsal midline thalamus in healthy volunteers [56] and proposed its role in the BSAR. However, in the absence of any causal connection, this remains an intriguing finding. Therefore, while it is clear that these cortical and sub-cortical structures do participate in the BSAR, their precise role remains undefined. 

### 2.5 Concluding Remarks

It is clear that despite the involvement of a variety of factors, the focus of each of these hypotheses has been relatively narrow. Not surprisingly, none of these hypotheses satisfactorily explain the BSAR. Evidence for the increased monocarboxylate fuel hypothesis, the refined brain glycogen super-compensation hypothesis and the role of higher brain structures is indirect. This provides no insight into the causal relationship among these factors that causes the BSAR. Much of the evidence for the hypothesis that the BSAR occurs due to changes in the sensitivity of GSNs in the VMH is not only indirect but also conflicting and ambiguous. Furthermore, an additional caveat in this hypothesis is that it implicitly assumes that the VMH is the hypothalamic centre that primarily mediates and regulates the AR against clinical hypoglycaemic events (that usually involve a slow prolonged fall in glucose) [57-59]. This is a questionable assumption. This is because the suggestion of the role of the VMH is mainly derived from an earlier set of studies– through the use of experimental techniques such as lesion and local infusion of 2-DG and glucose [27-29] – and the experimental protocol that involves repeated injections of insulin which induce rapid decline in glucose that are sustained for prolonged periods of time, i.e., ~2 hours [57-59]. These experiments while suggesting the role of VMH in the SAR, have masked the underlying complexities and subtleties involved in the AR (including during insulin-induced hypoglycaemia), which is also relevant to this clinical problem as described below. 

Investigators showed that in a number of situations such as fasting and hypoglycaemia (without exogenous insulin-administration), the adrenal medulla is exclusively stimulated while the SNS is suppressed [3, 60]. This suggests that dissociation occurs in the sympathetic and adrenal response. This is also consistent with the finding that in the fasted animals, the hormonal response against insulin-induced hypoglycaemia is mainly mediated by the AR [61]. In this regard, investigators showed that the LH mediates the AR in the fasted state [82]. Furthermore, portal vein glucose sensors sense slowly developing insulin-induced hypoglycaemia [57-59], and during the rapid glucose decline, the critical locus for hypoglycaemic detection shifts away from the portal-mesenteric vein to another central loci (e.g., presumably to the VMH) [57]. Since the portal vein sensors send afferents to the LH [62], this also suggests that the LH mediates the AR during slowly developing hypoglycaemia. Indeed, this is compatible with a converging line of evidence that suggests that a hierarchical network of glucose-sensitive neurons which connects the LH, NTS and the hepatic portal vein, also mediates the AR [63]. This network operates with redundancy–if the glucose-sensitive neurons in the LH are inactivated, the AR would still be elicited, albeit with variable magnitude, through the other glucose-sensitive neurons (i.e. in the NTS) [63]. Since the majority of clinical hypoglycaemic events develop slowly rather than rapidly [57, 64] and since the portal vein participates in such hypoglycaemia episodes [57-59], this suggests that the mechanism that inhibits activity of glucose-sensitive neurons in the LH causes the blunting of the AR. 

This evidence, nevertheless, does not dilute the potential importance of GSNs, located in the VMH, in the BSAR. In fact, earlier work of Oomura *et al*. [65] showed that a reciprocal relationship exit between these glucose-sensitive neurons in the LH and the VMH. Later, Arees and Mayer demonstrated the anatomical connections between these two hypothalamic sites, providing an anatomical basis for this relationship [66]. Consistent with this, evidence has recently emerged that reciprocal GABAergic projections exist between the VMH and the LH [67, 68]. Evidence also exists to suggest that the LH facilitates the AR, whereas the VMH inhibits it [69, 70]. Altogether, these findings suggest that through GABAergic neurons the VMH regulates the AR, whereas glucose-sensitive neurons in the LH mediate it. Hence, BSAR could be the result of higher inhibitory tone downstream of the GSNs to the LH, but not due to the changes in the sensitivity of GSNs. 

What is clear from this review is that a new theory that can coherently integrate diverse evidence and suggest how a variety of factors interact to cause the BSAR is needed. Any such theory should also be able to explain the following observations:

- Dissociation in the blunting of the AR and the cholinergic sweating response (mediated by the SR) [10, 11, 71].
- Sexual dimorphism in the blunting of the AR [72].
- Defective awakening [1] and loss of vigilance [145].
- Blunting of the AR due to sleep and antecedent exercise [1].

The following part of the review describes a new theory, which explains the above observations. 

## 3 THE BRAIN STRUCTURES AND NEURAL CIRCUITRY INVOLVED IN THE SAR

The description of brain structures and neural circuits mediating the SAR is essential for understanding of the mechanisms that cause the BSAR. Therefore, the next section links these neural circuits and brain structures. 

### 3.1 The Brain Structures and Neural Circuitry Involved in the AR

The LH is involved in the AR. Electrical stimulation of the LH increases efferent activity of the adrenal nerves and evokes the AR [73, 74]. Data suggests that the glucose-sensitive orexin neurons, in the LH, are the loci of the AR [75]. Some of these neurons change their firing in inverse proportion to the local changes in glucose levels, whereas some of them are indirectly regulated by peripheral glycaemic signals [76-78]. Furthermore, Cai *et al*. discovered that hypoglycaemia causes a 10-fold increase in orexin-B levels [77]. Matsumura *et al*. found that the stimulation of these neurons through central administration of orexin-A increases epinephrine secretion [79]. Nijima *et al*. showed that portal vein glucose infusion reduced efferent activity in adrenal nerves, [80, 81] suggesting reduction in epinephrine secretion. Since orexin neurons are inhibited by the rise in portal vein glucose and feeding [77, 78], this evidence clearly suggests orexin neurons mediate the AR. Collectively, data suggest that orexin neurons in the LH form a central locus of the AR. 

How does hypoglycaemia-induced excitation of orexin neurons evoke the AR? The hippocampal-LH cholinergic neuronal axis mediates the AR. Specifically, activation of the muscarinic receptor in the hippocampus causes hyperglycaemias through the AR in the fasted state [82, 83]. Hippocampal cholinergic neuronal activity is then transmitted to the LH cholinergic neurons which mediate the AR [82, 83]. Since orexin neurons are active during the fasted state and since they mediate the AR [82], this suggests that orexin neurons may stimulate the hippocampal cholinergic neurons involved in the AR. Indeed, Wu *et al.* showed that orexin neurons innervate the septal-hippocampal area and excite hippocampal cholinergic neurons through orexin-2 receptors [84]. This suggests that orexin neurons excite the Hippocampal-LH cholinergic neuronal axis and evoke the AR. 

How does acetylcholine (ACh) cause the AR? Data suggests that CRF neurons in the PVH [45] and PGF_2_α mediate the AR [85]. From these facts and by the analogy to acetylcholine-mediated vasopressin release in the PVH [86], a mechanism can be suggested as to how ACh release causes epinephrine secretion. ACh secretion activates intra-hypothalamic cholinoceptive intraneurons (IIN) (that connects the LH and PVH) which causes the release of NO from them [86, 87]. This NO diffuses to the CRF neurons, and activates the cyclooxygenase enzymes which generate PGF_2_α, in turn activating CRF neurons and causing CRF secretion which activates CRF-1 receptors [87]. This leads to the release of thromboxane-A_2_, another eicosanoid, through NO [87, 88]. Conceivably, through some hitherto unknown neurons, thromboxane-A_2_ in turn stimulates descending catecholaminergic neurons in the RVLM (C1 group), which projects to the preganglionic neurons, stimulating the adrenal medullae to secrete epinephrine [89, 90]. 

**Fig. (1):**
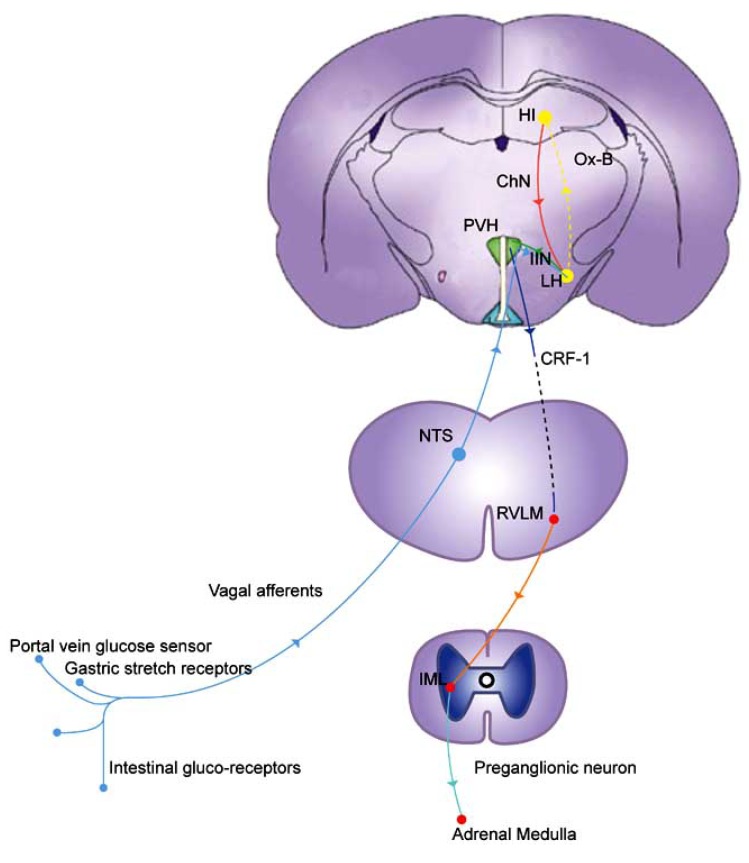
**Brain regions and afferent and efferent neural circuitry involved in the AR.** Hypoglycaemia is first detected at the peripheral glucose sensors. Through the vagal afferents this information is relayed to the orexin neurons in the LH *via* the NTS. In turn, through Orexin-B receptors, orexin neurons excite the hippocampal- LH cholinergic (ChN) neuronal axis. Through muscarinic receptors, cholinergic excitation in the LH in turn activates (some as yet unknown type of) IIN which then activates a cascade in the PVH involving the eicosanoids (thromboxane-A2 and prostaglandin) and the CRF-1. Activation of this cascade excites descending catecholaminergic neurons in the RVLM (C1 group), which projects to the preganglionic neurons that stimulate the adrenal medullae to secrete epinephrine. (---) lines indicate peptide neurotransmitters. (Modified and reproduced with permission from Silveira *et al.* [90] and Barsh et al. [158]).

In summary, orexin-induced stimulation of the hippocampal-LH axis causes acetylcholine release in the LH. ACh release in turn, through the intrahypothalamic interneuron, activates a cascade in the PVH involving the eicosanoids and CRF (as illustrated in Fig. **1**). Through stimulating catecholaminergic neurons in the hindbrain, this cascade ultimately causes the AR. 

### 3.2 The Brain Structures and Neural Circuitry Involved in Anxiety and the SR

Hypoglycaemia causes anxiety which, in this context, is an unpleasant homeostatic emotion related to an *introceptive *threat accompanied by negative mood changes [91]. Anxiety can occur unconsciously [92]. It is also accompanied by both behavioural and physiological responses including sweating or sympathetic skin responses (SSR), arousal and attention bias [91, 93]. 

The brain circuits and areas mediating the homeostatic emotions, due to *introceptive* threats, are now beginning to be revealed and defined. Studies implicate the cortical structures such as the OFC, ACC and insula. Sub-cortical structures such as the amygdala, hypothalamus and brainstem have also been implicated [94-98]. The brain imaging studies have shown that hypoglycaemia also activates these structures [51, 52, 54-56]. 

Among these structures, a dynamic coalition of the amygdala and OFC is the principal determinant in the generation of anxiety [99, 100]. The amygdala generates anxiety and associated responses as well as recruits higher cortical structures (i.e. the insula) as part of an early alerting system for biological salient signals (fear), without conscious awareness [99-103]. By modulating the amygdala’s activity, the OFC regulates emotions and associated responses [99, 100]. The OFC areas issue a dense glutamatergic projection to the posterior part of the amygdala that includes the basolateral nucleus and intercalated nuclei which are clusters of GABAergic inhibitory neurons [99-101]. Thus, the glutamatergic OFC projection modulates the amygdala’s activity and regulates emotions through modulating GABAergic neuron activity in the amygdala [99-101]. Consistent with this, Dunn *et al. *also showed that, compared to hypoglycaemic aware subjects, the subjects with HU showed an increase in lateral OFC (lOFC) activity and a reduction in amygdala activity [55]. This raises the possibility that a mechanism that increases the lOFC activity (which has direct connections with the amygdala [104]) could be responsible for HU. 

The relevant afferent glycaemic signals to these brain areas are relayed by lamina I neurons of the spinal dorsal horn via the NTS and PBn [98]. The neurons from the PBn, in turn, convey this information to the amygdala, ACC, insula and the OFC [105]. Moreover, glucose sensors in the hepatic portal vein, gastrointestinal area and gastric stretch receptors also send relevant information through the vagal afferents to these areas through the NTS [105]. 

## 4 A HEURISTIC THEORY ON THE MECHANISMS OF THE BSAR

Based on the earlier experiments that suggest two mechanism of the BSAR [10, 11, 71], this theory suggests two mechanisms. Part of this theory which explains AR blunting builds on two previous hypotheses: the complementary role of brain glycogen and lactate [1]. Similarly, this theory agrees with Dunn *et al*. that receptor de-sensitisation mediates the blunting of the SR and causes HU [55]. 

### 4.1 The Mechanism of the AR Blunting: the Role of NTS (A2 group) Noradrenergic Neurons

GABAergic neurons are widespread in the CNS, including the hypothalamus, and play an inhibitory role to regulate neurohormonal responses including the AR [83]. Indeed, data indicates that the GABAergic interneuron modulates the AR through the GABA_A_ receptor mechanism [83]. The GABAergic neurons project from the VMH to the LH [67, 68]. This suggests that increase in GABAergic ‘tone’ in the VMH, due to repeated hypoglycaemia, inhibits the activity of orexin neurons and blunts the AR. 

#### Recurrent NA Release Increases GABAergic Tone

A rise in GABAergic ‘tone’ requires a rise in GABA synthesis and its secretion*.* Lactate mediates an increase in GABA synthesis and its release. Waagepetersen *et al.* demonstrated that GABA synthesis is compartmentalised and involves a mitochondrial TCA cycle in the GABAergic neurons [106]. They found that the compartment which mediates GABA synthesis is related to the mitochondria TCA cycle that preferentially metabolises lactate as a substrate [106]. Duarte *et al.* demonstrated that monocarboxylate fuels alone are capable of sustaining sufficient vesicular filing with GABA [107]. Further, monocarboxylate supported GABA release is two fold higher than that supported by glucose, suggesting high GABAergic turnover [108]. Altogether, these findings suggest that repeated hypoglycaemia somehow increases lactate supply to GABAergic neurons thus raising the synthesis and release of the GABA. 

By up-regulating the expression of MCT2, NA mediates the increases in lactate supply to GABAergic neurons [109]. Moreover, NA also mediates lactate release from the astrocytes (through glycogenolysis), the slow process of glycogen re-synthesis and glucose entry into the astrocytes [12]. Thus, through three processes NA mediates the blunting of the AR. Indeed, researchers showed that NA neurons from the hindbrain hyperpolarise orexin neurons through GABAergic neurons [110, 111]. Furthermore, in keeping with this theory, hypoglycaemia causes concomitant rise in both NA and GABA levels in the VMH [112]. Of note, this release of NA is not affected by recurrent hypoglycaemia. NA neuronal groups, A1 and A2, from the hindbrain respond to glucoprivation and project to the medial hypothalamus [113]. Since electrical stimulation of the A1 neuronal group increases NA and GABA in the POA of the hypothalamus [112], it is likely that the A2 group provides the source of NA. This theory would predict that a lesion of the NA neurons should increase the AR. Indeed, the lesion of central noradrenergic pathways induces adrenal hyperactivity in rats [114]. Interestingly, Simizu *et al*. recently found that the central mechanism of sodium regulation also uses a similar mechanism [115]. 

#### GI Neurons in the VMH Mediates the NA Release During Hypoglycaemia 

This theory raises an interesting question: How does hypoglycaemia cause NA release in the VMH? Data suggests that NA release in the VMH inversely depends on local glucose levels within the VMH [113]. Investigators found that cholinergic neurons stimulate the NA neurons to release NA in the VMH [116]. Since glucose levels, through GI neurons, inversely regulate NO levels [117], and since NO causes ACh release [118], this suggests that during hypoglycaemia, through NO, GI neurons activate a neuronal cascade involving cholinergic, noradrenernaline and GABAergic neurons in the VMH which mediate the process that ultimately blunts the AR. 

**Fig. (2):**
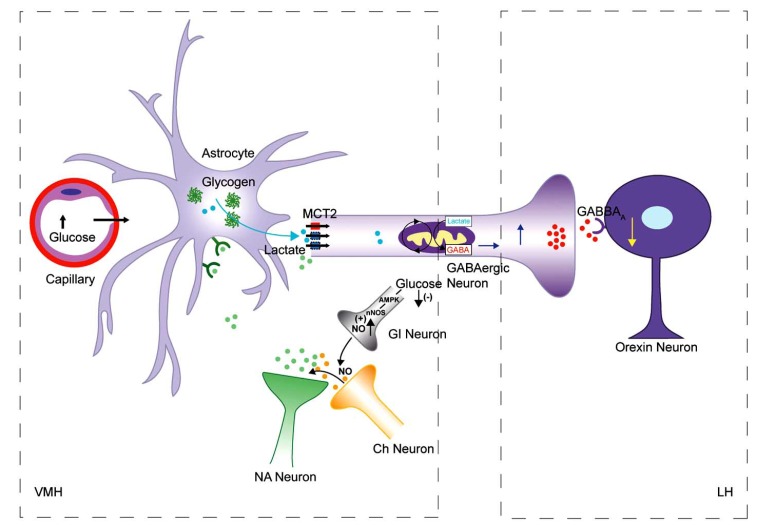
**Model of the blunting of the AR.** An axon terminal of an NA neuron from the hindbrain is shown. NA release from this terminal regulates 1) the expression of monocarboxylate transporters, MCT2, and mediates the glycogenolysis, glycogen-resynthesis and glucose entry into the astrocytes. This model suggests that during an episode of hypoglycaemia, low glucose levels increase the release of NO from GI neurons. Higher NO levels, *via* cholinergic (Ch) neurons, increase NA release from the axon terminals of the noradrenergic neurons (A2 group) in the VMH, which projects from the hindbrain. This NA release in the VMH up-regulates MCT2 expression (shown blue). Consequently, during a later episode of hypoglycaemia when, again, NA is released from these axon terminals, more lactate enters into the GABAergic neurons (which project to the LH). This leads to higher GABA synthesis and release, causing hyperpolarisation of orexin neurons, through GABA_A_ receptors, and thus blunts the AR. (Modified and reproduced with permission from Magistretti and Allaman [119], Iadecola [115], and Canabal et al. [117]).

In summary (as illustrated in Fig. **2**) this theory posits that hypoglycaemia causes GI neurons to release NO. Through cholinergic neurons, NO facilitates NA release from the NA neurons that project from the hindbrain. Frequent NA release, due to recurrent hypoglycaemia, up regulates the MCT2 transporters, increasing the supply of lactate to GABAergic neurons. This ultimately leads to higher GABAergic tone in the VMH. Through GABAergic projection to the LH from the VMH, an increase in GABAergic tone ultimately suppresses orexin neurons in the LH and blunts the AR. 

### 4.2 The Mechanism of HU: De-Sensitisation of β-Adrenoreceptors (β-AR) in the lOFC

The brain noradrenergic system regulates anxiety and associated behavioural and physiological responses such as attention, vigilance, arousal, mood and the SSR [120-124]. NA release, specifically from the locus coeruleus--noradrenergic (LC-NA) neurons, into the cortex causes anxiety and associated responses [124, 125]. Indeed, higher brain NA levels in the OFC are correlated with anxiety [126]. Further, intake of caffeine and other similar methylxanthine compounds – that increase NA release in the brain by activating LC-NA neurons –increase anxiety and associated responses [127-129]. This is also in keeping with the fact that coffee ingestion increases hypoglycaemia awareness [130]. Taken together, these findings suggest that the brain noradrenergic neurons mediate emotions related to threat and associated responses. 

Hypoglycaemia activates LC-NA neurons [131-133], suggesting that LC-NA neurons induce anxiety and associated responses during an episode of hypoglycaemia. This also suggests that repeated hypoglycaemia modifies the sensitivity of noradrenergic system in the cortex and causes HU. Stressor-induced rapid and reversible receptor desensitisation of the adrenergic receptor is such a mechanism [133]. Indeed, this mechanism is well described in animal models of stress adaptation in which chronic stress induces adaptation in the behavioural and physiological responses through changes in the sensitivity of β-ARs in the cortex [134-136]. Thus, conceivably repeated exposure of NA from LC-NA neurons, due to frequent hypoglycaemia, induces βAR desensitisation in the lOFC and causes HU. 

How do anxiety and its associated responses occur during hypoglycaemia? Hypoglycaemia activates the LC-NA neurons [131-133] which project to the lOFC [104] and excite GABAergic neurons through post-synaptic β-ARs [137]. These GABAergic neurons, in turn, hyperpolarise the glutamatergic neurons in the lOFC [137] and reduce glutamatergic neuronal transmission to the amygdala [99-101,  137]. Since glutamatergic neurotransmission from the lOFC inhibits the amygdala’s activity through activation of GABAergic neurons [99-101, 126], high NA level-induced hyperpolarisation of glutamatergic neurons in the lOFC facilitates the generation of anxiety and associated responses. 

How does the receptor de-sensitisation occur that leads to HU? The following events are proposed: an intermediate or prolonged episode of insulin-induced hypoglycaemia [11] raises NA levels in the lOFC. Hypoglycaemia-induced high NA levels cause β-AR desensitisation in the lOFC. Consequently, due to such repeated hypoglycaemic episodes, GABAergic mediated inhibition of glutamatergic neurons is progressively reduced in the lOFC. This causes a corresponding increase in the glutamatergic excitation of GAB Aergic neurons in the amygdala, reducing the amygdala’s activity. Thus anxiety and the SSR are progressively blunted leading to HU [55]. 

Since receptor de-sensitisation is reversible, this theory would predicate that manipulations that deprive β-ARs from the repeated exposure of NA would increase the β-AR sensitivity and thus would reverse HU. Indeed, studies have shown that in both healthy and T1DM hypoglycaemia unaware patients, antecedent use of three beta-blocker drugs for a week or more increased sweating [138, 139]. This also explains why scrupulous avoidance (2-3 weeks) of hypoglycaemia in T1DM patients causes reversal of HU [1]. The various antidepressant drugs exert their clinical effects by de-sensitizing the brain’s adrenergic receptors [134, 140]. This fact also explains why anti-depressant medication in some T1DM patients causes HU [130]. Furthermore, receptor sensitivity is affected also by the transmitter level which accumulates at the postsynaptic membrane [141]. Therefore, even a single but prolonged episode, which would cause high NA release, could also produce β-AR de-sensitisation and thus HU. 

## 5 SEXUAL DIMORPHISM AND ANTECEDENT EXERCISE-INDUCED PARTIAL BSAR

This theory explains the sexual differences in the blunting of the AR and antecedent-exercise induced blunting of the AR to subsequent episodes of hypoglycaemia or exercise [72]. 

### 5.1 The Cause of Sexual Dimorphism

Researchers reported a higher magnitude of the AR in men compared to women during an acute episode of hypoglycaemia and the relative preservation of the AR in women compared to men after antecedent hypoglycaemia. Vavaiya *et al*. found that oestradiol/oestrogen mediates regulation of MCT2: during an acute episode of hypoglycaemia it up-regulates MCT2 expression, whereas, during repeated episodes it down-regulates MCT2 expression [142]. Therefore, during an acute episode of hypoglycaemia in women, more lactate enters GABAergic neurons, whereas during repeated hypoglycaemia less lactate enters into these neurons. Thus, in women, GABAergic inhibition during acute and repeated hypoglycaemia is enhanced and diminished, respectively, relative to men. Therefore, women have a smaller magnitude of the AR during an acute episode but better defences against repeated hypoglycaemia [72]. 

### 5.2 Antecedent Exercise-Induced Partial BSAR

Investigators reported that antecedent hypoglycaemia blunts the AR to subsequent exercise. Similarly, antecedent bout of exercise blunts the AR to subsequent hypoglycaemia [1]. Since the NA neuronal system also mediates the AR during exercise [143] and since exercise also stimulates the hindbrain NA neuronal groups [144], this clearly suggests that antecedent-exercise activates the same mechanism thus explaining why antecedent hypoglycaemia and exercise reciprocally blunt subsequent AR. 

## 6 DISSOCIATION OF THE BLUNTING OF THE AR AND SR

This theory posits a role of LC-NA neurons in the BSAR. The involvement of LC-NA neurons provides the reasons for the different time course or dissociation of the blunting of the AR and SR [10, 11, 71]. Data suggests that intermediate and prolonged episodes of hypoglycaemia (in which the nadir of 2.9 +/- 0.1 mmol was reached in 30 mins and kept at this level for another 30 mins and 90 mins respectively), but not a short duration of hypoglycaemia blunts the symptoms [11]. The timing of the blunting of the symptoms is consistent with the time course of activation of LC-NA neurons in the brain [131, 132]. For example, Morilak *et al*. showed that changes in LC-NA neurons in cats occurred 30-60 mins following insulin administration, whereas the AR occurred within 15 mins [132]. Thus, the latency of LC-NA neuronal response explains why intermediate or prolonged episodes of hypoglycaemia, but not short episodes, cause HU. 

## 7 SLEEP AND HYPOGLYCAEMIA 

Hypoglycaemia, often with higher severity, commonly occurs during sleep in T1DM patients, [1, 145, 146]. Thus, hypoglycaemia during sleep poses a major problem. This is due to the additive effect of (1) the blunting of the AR during sleep [1, 147] and (2) repeated hypoglycaemia-induced defective awakening [148]. 

### 7.1 The Blunting of the AR During Sleep 

Gais *et al. *found that at the same time of night compared to being awake, being asleep *per se* shifts the onset of the AR to hypoglycaemia by about 0.6 mmol/l towards lower glucose levels [149]. This indicates that light influences the AR. Indeed, researchers showed that light amplifies the activity of the adrenal nerve [150]. In this regard, evidence is emerging that light influences the adrenal nerve through melatonin [151]. Niijima *et al. *showed that intravenous melatonin infusion suppresses the adrenal nerve’s activity [151]. This suppressive effect of melatonin seems to be mediated by afferent signals from melatonin sensors in the hepatoportal region [151]. This is consistent with evidence that glucose-sensitive neurons in the LH, NTS and the hepatic portal vein mediate the AR. Thus, light-induced alteration in melatonin levels might explain why sleep per se blunts the AR. However, further studies are required in this direction. 

### 7.2 The Cause of Defective Wakening

Paranjape *et al.* discovered that repeated episodes of prolonged hypoglycaemia cause the functional habituation of orexin neurons containing orexin-A [152]. Since these neurons, which are located in the perifornical and dorsomedial hypothalamus [153, 154], mediate arousal and wakening [75], functional habituation causes defective awakening in T1DM patients [1]. Furthermore, since these neurons mediate vigilance [155], habituation of these neurons explains why loss of vigilance [156] occurs in these patients.

## 8 THERAPEUTIC MEASURES

From this theory, the following therapeutic measures can be suggested to restore 1) the AR, 2) symptom response and 3) improving vigilance and waking response to hypoglycaemia. The AR can be restored by manipulating the brain glycogen levels. So, it follows from this that high intensity exercise, which depletes brain glycogen, could be prescribed to achieve controlled brain glycogen depletion as a therapeutic measure to overcome the blunting of the AR [15-17]. An additional advantage of this approach is that it has none of the side-effects that might accompany traditional pharmacological approaches such as the use of GABA_A_ receptor blockers. Discrete use of beta-blockers, which increase the β-AR sensitisation, can be employed to restore sweating– a critical symptom in recognition of hypoglycaemia. Furthermore, since orexin-A neurons are involved in the serious problems of defective awakening and loss of vigilance, these problems may be treated with nasal delivery of orexin-A [157]. 

## 9 CONCLUSION AND SUMMARY

The BSAR, due to repeated hypoglycaemia, causes HU. HU is responsible for higher morbidity and mortality in T1DM patients [1]. Obviously, this problem has led to intense research efforts and generated many hypotheses. 

I propose a new heuristic theory that attempts to integrate diverse lines of experimental data and clinical observations to satisfactorily explain the BSAR. Experimental validation for this theory awaits further studies and, no doubt, that some modifications will be needed in this theory when new facts will emerge. Nevertheless, it is hoped that the overall theoretical framework will hold and prove valuable in solving this clinical problem. The theory can be summarised as follows:

- During an episode of hypoglycaemia, low glucose levels increase the release of NO from GI neurons. Higher NO levels, *via* cholinergic neurons, increase NA release from the axon terminals of the noradrenergic neurons in the VMH. NA mediates the slow process of glycogen re-synthesis, rapid glycogenolysis and glucose entry into the astrocytes. Consequently, after an episode of hypoglycaemia when normoglycaemia is restored, this NA restores normal glycogen levels in the astrocytes within a few hours. Concomitantly, this NA release increases the MCT2 expression levels.
  During a later hypoglycaemic episode, when NA is released again in the VMH, lactate is released from the astrocytes. Since MCT2 numbers have increased, more lactate enters the neurons during this episode. This increased availability of lactate causes higher GABA synthesis, turn-over and release in the VMH. This rise in GABAergic ‘tone’ inhibits the orexin neurons in the LH through GABAergic connections from the VMH to the LH thus blunting the AR.
- An intermediate or prolonged episode of insulin-induced hypoglycaemia raises NA levels in the lOFC. This causes a de-sensitisation of β-AR receptors in the OFC. Such repeated hypoglycaemic episodes cause frequent high NA levels and further de-sensitises and down regulates these receptors. Consequently, the GABAergic mediated suppression of glutamatergic neurons is progressively reduced in the lOFC, causing a corresponding increase in glutamatergic excitation of GABAergic neurons in the amygdala. This reduces the amygdala’s activity. As a result, anxiety and associated responses are reduced, leading to HU.
  The role of orexin neurons containing orexin-A peptide and sleep-induced melatonin secretion is proposed to explain defective awakening and blunting of the AR during sleep respectively. Also, therapeutic measures have been suggested that will reduce or prevent severe episodes of hypoglycaemia, a major hindrance to insulin therapy thus improving the quality of life for millions of diabetic patients.

## ABBREVIATIONS

ACh: = Acetylcholine
AMPK: = AMP-activated protein kinase
AR: = Adrenomedullary response
ARC: = Arcuate
BSAR: = Blunting of the sympatho-adrenal response
Ch: = Cholinergic
CRF: = Corticotrophin-releasing factor
CRF-1: = Corticotrophin-releasing factor-1
CRF-2: = Corticotrophin-releasing factor-2
2-DG: = 2-deoxy-D-glucose
GABA: = Gamma-aminobutyric acid
GE: = Glucose-excited neuron
GI: = Glucose-inhibited neuron
GK: = Glucokinase
HU: = Hypoglycaemia unawareness
IIN: = Intrahypothalamic (cholinoceptive) intraneurons
K_ATP_: = ATP-sensitive potassium channel
LH: = Lateral hypothalamus
MCT2: = Neuronalmonocarboxylate transporter 2
mPFC: = Medial prefrontal cortex
NO: = Nitric oxide
NA: = Noradrenernaline
NTS: = Nucleus of the solitary tract
OFC: = Orbitofrontal cortex
PBn: = The parabrachial nucleus complex
PGF_2_α: = Prostaglandin F_2_α
PVH: = Paraventricular nucleus of hypothalamus
POA: = Preoptic area
rACC: = Rostral anterior cingulated cortex
RVLM: = Rostral ventrolateral medulla
SAR: = Sympatho-adrenal response
SR: = Sympathoneural response
T1DM: = Type 1 diabetes mellitus
T2DM: = Type 2 diabetes mellitus
VMH: = Ventromedial hypothalamus
SNS: = Sympathetic nervous system
SSR: = Sympathetic skin response
TCA: = Tricarboxylic acid
